# Diversity is the spice of life: An overview of how cytokinesis regulation varies with cell type

**DOI:** 10.3389/fcell.2022.1007614

**Published:** 2022-11-07

**Authors:** Imge Ozugergin, Alisa Piekny

**Affiliations:** ^1^ Department of Biology, McGill University, Montreal, QC, Canada; ^2^ Department of Biology, Concordia University, Montreal, QC, Canada

**Keywords:** mitosis, cytokinesis, RhoA, actomyosin, mitotic spindle, chromatin

## Abstract

Cytokinesis is required to physically cleave a cell into two daughters at the end of mitosis. Decades of research have led to a comprehensive understanding of the core cytokinesis machinery and how it is regulated in animal cells, however this knowledge was generated using single cells cultured *in vitro*, or in early embryos before tissues develop. This raises the question of how cytokinesis is regulated in diverse animal cell types and developmental contexts. Recent studies of distinct cell types in the same organism or in similar cell types from different organisms have revealed striking differences in how cytokinesis is regulated, which includes different threshold requirements for the structural components and the mechanisms that regulate them. In this review, we highlight these differences with an emphasis on pathways that are independent of the mitotic spindle, and operate through signals associated with the cortex, kinetochores, or chromatin.

## Introduction

### Overview of cytokinesis in animal cells

Cytokinesis must occur with high fidelity to prevent pathologies, and multiple pathways create a robust system to accommodate perturbations. While the relative role of these pathways likely varies with cell fate, ploidy and size, we lack knowledge of how they function in most cell types and tissues. Since several reviews describe the core cytokinesis machinery in depth, we will emphasize differences in cytokinesis among animal cell types (e.g., Green et al., 2012; Basant and Glotzer, 2018; Leite et al., 2019; Pintard and Bowerman, 2019; Pollard and O'Shaughnessy, 2019; Nguyen and Robinson, 2020; Sugioka, 2022).

**FIGURE 1:**
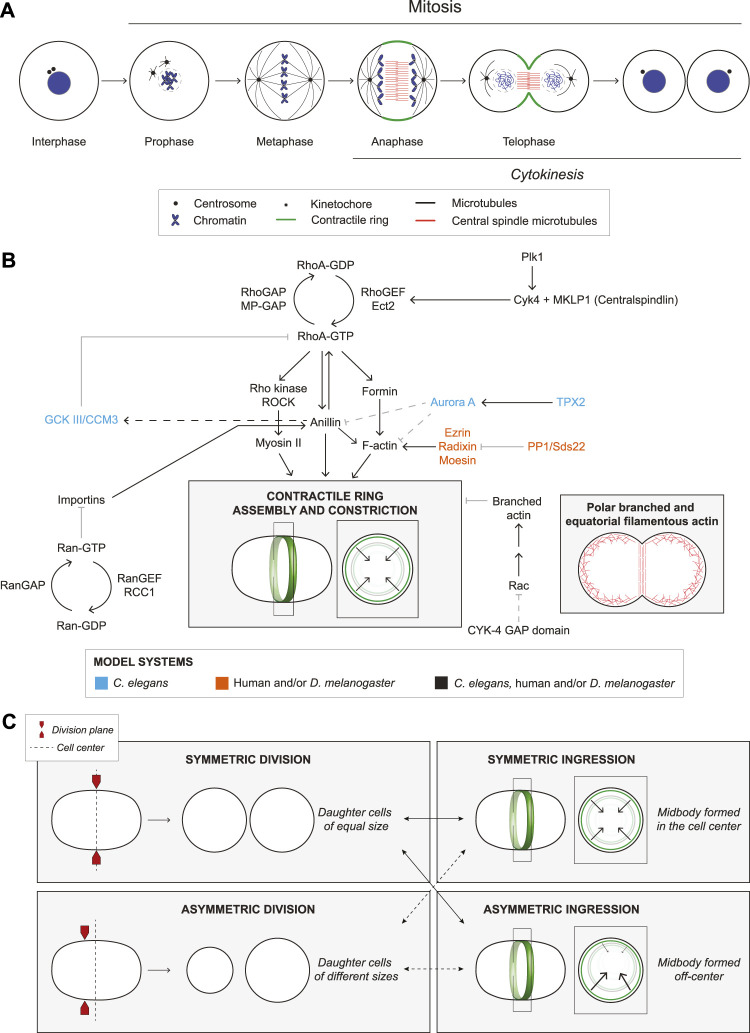
The molecular regulation of ring assembly and constriction. **(A)** The cartoon schematic shows a cell undergoing mitosis. Chromosome condensation and nuclear envelope breakdown occur during prophase. In metaphase, centrosomes (black circles) form a bipolar spindle (black) that aligns the sister chromatids (blue). During anaphase, the central spindle forms, consisting of anti-parallel bundled microtubules (red). A contractile ring (green) assembles between the segregating chromosomes and in a plane that bisects the central spindle. During telophase, the ring constricts to divide the cytosol, and the nuclear membrane reassembles. After the ring ingresses, a midbody forms that controls abscission to separate the two daughter cells. **(B)** Multiple proteins regulate cytokinesis as indicated by the arrows (solid lines are established interactions, while dashed lines are hypothetical). These pathways culminate in the assembly and constriction of an actomyosin ring (cartoon cell, ring in green). To the right, another cell shows the location of polar, branched F-actin (red branches) and linear F-actin (red lines) during cytokinesis. Font colors indicate whether studies were performed in *C. elegans* (blue), human cells and/or *D. melanogaster* (orange), or all three (black). **(C)** Cartoon schematics show cells undergoing symmetric division (top left), where two daughter cells of equal sizes are generated, and an asymmetric division (bottom left) forming daughter cells of different sizes. In either type of division, the ring can ingress symmetrically (top right) or asymmetrically (bottom right) where there is more ‘pull’, from one side of the ring.

**FIGURE 2:**
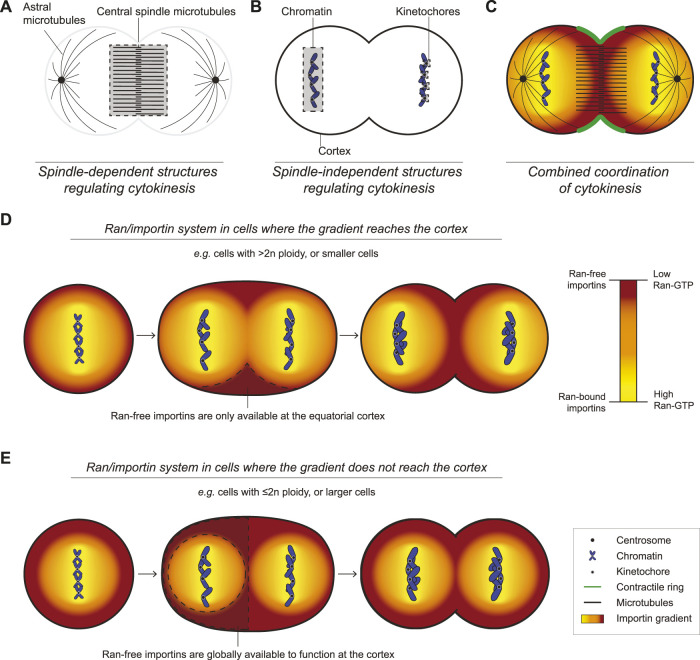
An overview of the mechanisms regulating cytokinesis. **(A)** A cartoon cell shows the spindle components relative to the overlying cortex in late anaphase/early telophase. **(B)** A similarly staged cell shows the relative locations of the chromatin, kinetochores, and cortex. **(C)** All of the key components from **(A)** and **(B)** are shown together in a one cell, which work together to ensure successful cytokinesis (Ran-free importins in dark orange, Ran-GTP in yellow, scale below). **(D)** Cartoons show how in small cells or in cells with high ploidy, Ran-GTP could restrict cortical importins, which only reach sufficient levels to recruit anillin to the equatorial cortex during anaphase as a dominant mechanism to control ring positioning. **(E)** Cartoons show how in large cells or in cells with low ploidy, Ran-GTP may not reach the cortex and importins would be able to recruit anillin uniformly to the cortex in metaphase and anaphase. These cells would require other mechanisms to control ring positioning. The legend indicates the relevant components for all cells **(A–E)**.

Cytokinesis occurs by the ingression of an actomyosin ring that constricts to pinch in the membrane (Figure 1A). The anaphase spindle provides cues for RhoA-dependent ring assembly in the equatorial plane (Figure 1B and Figure 2A; Rappaport, 1986; Bement et al., 2005). RhoA-GDP is inactive, while RhoA-GTP binds to effectors including formins and Rho-kinase (ROCK) to generate linear actomyosin filaments (Figure 1B; Piekny et al., 2005; Green et al., 2012). The GTPase activating protein (GAP) MP-GAP (*Ce*RGA-3/4) globally inactivates RhoA by stimulating GTP hydrolysis, while the guanine nucleotide exchange factor (GEF) Ect2 (*Ce*ECT-2, *Dm*Pbl) activates RhoA by exchanging GDP for GTP (Figure 1B; Tatsumoto et al., 1999; Yuce et al., 2005; Zanin et al., 2013). Ect2 activity is spatiotemporally controlled by centralspindlin (Cyk4/MgcRacGAP, *Ce*CYK-4, *Dm*RacGAP50C and MKLP1/KIF23, *Ce*ZEN-4, *Dm*Pav), which bundles microtubules to form the central spindle during anaphase (Mishima et al., 2002; Yuce et al., 2005; Hara et al., 2006; Niiya et al., 2006). Cyk4-binding recruits Ect2 to the central spindle (Figure 1B; Yuce et al., 2005; Petronczki et al., 2007; Wolfe et al., 2009). Cyk4 also requires Plk1 phosphorylation for Ect2-binding, and the loss or inhibition of Plk1 or Cyk4, and/or blocking Cyk4 phosphorylation prevents ring assembly and phenocopies Ect2 depletion (Somers and Saint, 2003; Zhao and Fang, 2005; Burkard et al., 2007; Miller and Bement, 2009; Wolfe et al., 2009; Gomez-Cavazos et al., 2020). Plk1-phosphorylation could reduce the affinity of centralspindlin for microtubules, causing its release to the overlying membrane where it activates Ect2 and is regulated by Aurora B kinase (Petronczki et al., 2007; Wolfe et al., 2009; Frenette et al., 2012; Lekomtsev et al., 2012; Adriaans et al., 2019). RhoA-GTP also recruits anillin (*Ce*ANI-1), which crosslinks F-actin and myosin with phospholipids for ring positioning, and forms complexes with septins to facilitate ingression (Figure 1B; Piekny and Maddox, 2010; Carim et al., 2020). Anillin also feeds back to facilitate RhoA-GTP effector binding (Budnar et al., 2019). As linear filaments are generated in the equatorial plane, their alignment is facilitated by cortical flow and/or crosslinkers in the *C. elegans* zygote (Reymann et al., 2016; Khaliullin et al., 2018; Leite et al., 2020). Constriction then occurs by the myosin-dependent binding and/or sliding of actin filaments (e.g., Ma et al., 2012; Osorio et al., 2019). In addition, a hypothesis paper proposed that anillin-septin membrane microdomains are shed from the ring to relieve tension and mediate ring closure (Carim et al., 2020).

Despite our extensive knowledge of cytokinesis, studies suggest that the core structural components and their regulators do not play the same role in all cells. For example, differences in the organization, levels and threshold requirements of F-actin (e.g., Davies et al., 2018), myosin (e.g., Ozugergin et al., 2022), and formin (e.g., Davies et al., 2018; Higashi et al., 2019) would cause different cortical properties that affect ring closure kinetics (Leite et al., 2019).

## Differences in cytokinesis among animal cell types

Cytokinesis is influenced by intrinsic and extrinsic factors that affect filament alignment for constriction and include polarity, cell–substrate adhesion and adherens junctions (Higashi et al., 2016; Pinheiro et al., 2017; Dix et al., 2018; Chaigne et al., 2021; Gupta et al., 2021; Ozugergin et al., 2022; Paim and FitzHarris, 2022). Along with causing different rates of ingression, these factors can also cause ingression to be more asymmetric (Figure 1C). Here, we will describe differences in the core structural components and upstream regulators of the ring.

### Differences in structural ring components

Differences in the ring components can affect ring kinetics. Distinct actin and myosin isoforms can have different biochemical properties, while actin can form branched or unbranched filaments with different rates of assembly or disassembly. For example, distinct actin and myosin isoforms are differentially enriched in the equatorial plane compared to the polar cortex (Maupin et al., 1994; Dugina et al., 2009; Po’uha and Kavallaris, 2015; Chen et al., 2017; Yamamoto et al., 2019; Shagieva et al., 2020; Taneja et al., 2020; Chen et al., 2021). Different actins assemble into distinct linear or branched filaments *via* different formins or Arp2/3 (Figure 1B), while myosin isoforms have different crosslinking or motor activities (Bao et al., 2005; Chen et al., 2017; Taneja et al., 2020; Wang et al., 2020; Chen et al., 2021). In *C. elegans*, aligned actin filaments in the equatorial plane facilitate the assembly of new filaments (Li and Munro, 2021). The requirement for myosin’s function as a motor or crosslinker also differs between cell types in mice and *C. elegans* (Ma et al., 2012; Osorio et al., 2019). As mentioned earlier, levels could also affect ring kinetics. Partial depletion of ARX-2 (*Ce*Arp2) or CYK-1 (*Ce*formin) can alter ring dynamics by changing the levels of equatorial F-actin (Chan et al., 2019). Germline-fated cells in *C. elegans* embryos have less linear F-actin and myosin and slower ring assembly compared to somatic cells, and they operate closer to threshold requirements (Davies et al., 2018; Ozugergin et al., 2022). A prior study proposed that larger cells have more contractile units in the ring than smaller cells to coordinate ingression (Carvalho et al., 2009). However, ring closure has distinct phases that may or may not correlate with size (Davies et al., 2018; Ozugergin et al., 2022). The amount of actomyosin could cause different tension or flow rates that influence ring closure, which could be crucial during development. In *C. elegans*, signalling between P_2_ and EMS cells regulates their fate, and their relative positions are controlled by coordinating division at the two-cell stage (Rose and Gonczy, 2014; Davies et al., 2018).

### Differences in ring closure symmetry

Asymmetric ring ingression is more extreme in cells with apicobasal polarity or that contact other cells (Figure 1C). Symmetry breaking is modeled to occur through the positive feedback of membrane curvature-dependent filament alignment (Dorn et al., 2016). The mechanisms that control filament alignment could be influenced intrinsically or extrinsically as described earlier (Maddox et al., 2007; Singh and Pohl, 2014; Reymann et al., 2016; Spira et al., 2017; Khaliullin et al., 2018). Asymmetric alignment could cause higher contractility and/or different tension in part of the ring. However, the molecular regulation of asymmetric closure is not clear. CYK-1, ANI-1 and septins control asymmetric ingression in the *C. elegans* zygote (Maddox et al., 2007; Chan et al., 2019). However, in the vulval precursor cells, tissue geometry and adhesion play a stronger role (Maddox et al., 2007; Bourdages et al., 2014). PARD6B is required for apicobasal polarity and asymmetric ingression in the early mouse embryo, and the localization of anillin and myosin is mutually exclusive with apically-enriched PARD6B (Paim and FitzHarris, 2022). This mechanism differs from *Drosophila* epithelial cells where ingression is influenced by extrinsic forces transmitted through adhesion junctions (Herszterg et al., 2014; Osswald and Morais-de-Sa, 2019; Buckley and St Johnston, 2022).

### Differences in ring regulators

Differences in the upstream regulators can also affect ring kinetics. Ect2 and Pbl localize to microtubules and the equatorial cortex in HeLa cells, *Drosophila* embryos and S2 cells (Prokopenko et al., 1999; Yuce et al., 2005; Verma and Maresca, 2019), but ECT-2 is cortical in the *C. elegans* zygote (Gomez-Cavazos et al., 2020). Both Cyk4 and Ect2 require membrane localization to generate active RhoA for cytokinesis (Su et al., 2011; Frenette et al., 2012; Lekomtsev et al., 2012; Basant et al., 2015). Thus, the requirement for cortical centralspindlin and/or Ect2 could be higher in cells where the central spindle is far from the cortex. There is also a debate (Basant and Glotzer, 2017; Zhuravlev et al., 2017) about whether Cyk4 activates RhoA, or functions as a GAP for Rac. Point mutations that disrupt GAP activity cause cytokinesis phenotypes, and Rac depletion suppresses phenotypes caused by the loss of CYK-4 or ECT-2 in *C. elegans* embryos (Canman et al., 2008; Zhuravlev et al., 2017). CYK-4 was proposed to downregulate Arp2/3-mediated branched F-actin and decrease cortical stiffness in the equatorial plane (Figure 1B; Canman et al., 2008; Bastos et al., 2012; Zhuravlev et al., 2017). However, an alternative interpretation is that Rac globally regulates cortical stiffness and its depletion makes it easier for weakly formed rings to ingress (Loria et al., 2012; Basant and Glotzer, 2017). In HeLa cells, Cyk4 regulates RhoA, but it could also regulate Rac1 to control effectors for adhesion (Yuce et al., 2005; Bastos et al., 2012). Further research is needed to clarify the role of Cyk4 in cytokinesis in additional cell types.

Anillin also varies between cells. Anillin is cytosolic in interphase *C. elegans* and *Drosophila* embryonic cells, but is nuclear in cultured *Drosophila* and human cells (Piekny and Maddox, 2010). Anillin depletion causes cytokinesis failure in *C. elegans* neuroblasts, *Xenopus* embryos, *Drosophila* S2 and HeLa cells, but not in the *C. elegans* zygote, despite a ∼97% reduction in anillin levels (Maddox et al., 2005; Straight et al., 2005; Hickson and O'Farrell, 2008; Piekny and Glotzer, 2008; Piekny and Maddox, 2010; Fotopoulos et al., 2013; Reyes et al., 2014). Dalmatians with an early nonsense mutation in anillin were born, albeit with developmental defects, suggesting that anillin is not required for cytokinesis in most cells (Holopainen et al., 2017). However, alternative splicing, initiation codons or translation could still produce functional protein depending on the cell type. Anillin also plays multiple roles in cytokinesis, including ring positioning, ingression and midbody formation, which could require different threshold levels (Hickson and O'Farrell, 2008; Piekny and Glotzer, 2008). In the *C. elegans* zygote, ANI-1 controls ingression through negative feedback by recruiting GCK-1 and its cofactor CCM-3 to inactivate RhoA through RGA-3/4 for RhoA inactivation (Figure 1B; Rehain-Bell et al., 2017; Bell et al., 2020), while anillin controls RhoA-GTP signaling by facilitating its interaction with effectors in mammalian cells (Budnar et al., 2019). Anillin’s crosslinking function can also slide actin filaments and generate force *in vitro* without myosin (Kucera et al., 2021). The variable threshold requirements for anillin could reflect its different interactions and functions.

## Spindle-independent regulation of cytokinesis in animal cells

Spindle-independent pathways also regulate cytokinesis, and their requirement likely varies with cell fate, ploidy or size (Figure 2B). These pathways would contribute to the cytokinetic diversity of cells with different developmental paths, providing a robust system that precludes cytokinesis failure (Figure 2C).

### Cortical mechanisms

Aligned actomyosin filaments generate force for ring constriction. The ring forms within a continuous, cortical network that spans the cell, and actin-binding proteins that control cortical connectivity such as plastin and spectrin can influence this meshwork and stabilize the ring (Turlier et al., 2014; Ding et al., 2017; Leite et al., 2019; Sobral et al., 2021). Excess cytoplasmic pressure may arise in the polar cortex as the ring constricts, which is released by blebs that form from localized changes in the cortex (Sedzinski et al., 2011). For example, RhoA is typically inactive at the polar cortex, and blebs occur more frequently after MP-GAP depletion (Sedzinski et al., 2011; Zanin et al., 2013). Blebbing can vary among cell types, reflecting differences in their cortical properties; e.g., HeLa cells display more prominent blebbing than *C. elegans* embryos (Zanin et al., 2013).

Cortical pathways facilitate ring positioning in asymmetrically dividing cells (Figure 1C). *Drosophila* neuroblasts have apicobasal polarity and divide asymmetrically to produce daughter cells with different sizes and fates. The ring assembles closer to the basal pole where myosin enrichment is controlled by Pins and Dlg (Cabernard et al., 2010; Connell et al., 2011). In the *C. elegans* zygote, actomyosin contractility is enriched at the anterior cortex *via* feedback mechanisms that establish anterior-posterior polarity through the localization of distinct PAR (*par*titioning defective) complexes (Lang and Munro, 2017; Delattre and Goehring, 2021). The contractile ring aligns with the anterior-posterior boundary, but it is unclear how PAR proteins control ring position (Schenk et al., 2010; Pittman and Skop, 2012). One model is that anterior actomyosin competes for ANI-1, restricting its levels in the ring (Jordan et al., 2016).

### Chromatin sensing *via* kinetochores

Kinetochores regulate cytokinesis by promoting the removal of F-actin from the polar cortex (Figure 2B). Kinetochores are crucial for chromosome segregation by stably attaching chromosomes to the mitotic spindle (Musacchio and Desai, 2017; Lara-Gonzalez et al., 2021; Navarro and Cheeseman, 2021). Ezrin-Radixin-Moesin (ERM) proteins crosslink F-actin to the membrane to regulate cortical properties (Carreno et al., 2008; Kunda et al., 2008). As chromosomes segregate, kinetochore-associated PP1 phosphatase and Sds22 inactivate moesin, causing a decrease in polar F-actin in *Drosophila* S2 and HeLa cells (Figure 1B; Roubinet et al., 2011; Kunda et al., 2012; Rodrigues et al., 2015). While PP1/Sds22 and moesin are not required for cytokinesis, their depletion causes cell shape changes and membrane protrusions, respectively (Carreno et al., 2008; Rodrigues et al., 2015). The chloride channel CLIC4 also controls polar cortical stability through ezrin-binding, but it is not clear if CLIC4 is part of the kinetochore pathway (Peterman et al., 2020; Uretmen Kagiali et al., 2020).

Polar relaxation occurs through other mechanisms when kinetochores are far from the cortex. In *C. elegans* zygotes, astral microtubules regulate the polar cortex through AIR-1 (Aurora A kinase) and TPXL-1 (*Hs*TPX2), which inhibits the polar accumulation of ANI-1 and F-actin (Figure 1B; Mangal et al., 2018). More recent work in *C. elegans* revealed that astral microtubules control the dynein-dependent removal of myosin from the polar cortex (Chapa et al., 2020). Other studies showed that in *C. elegans* and cultured human cells, ANI-1/anillin binds to astral microtubules in cortical regions where RhoA-GTP is low, and astral microtubules cause a decrease in formin activity and γ-actin at the polar cortex (Tse et al., 2011; van Oostende Triplet et al., 2014; Chen et al., 2021). It is not clear if these mechanisms are related, and studies are needed to reveal how their requirement varies with cell type.

### Chromatin sensing *via* Ran signaling

Other chromatin sensing pathways regulate cytokinesis. Lagging chromosomes delay cytokinesis, likely to prevent aneuploidy (Steigemann et al., 2009; Kotadia et al., 2012; Montembault et al., 2017). In *Drosophila* neuroblasts, trailing chromatids correlate with broad myosin accumulation, cell elongation and delayed completion of cytokinesis (Kotadia et al., 2012). This phenotype is associated with delayed nuclear envelope assembly, leaving Pbl at the midzone where it could cause persistent RhoA activation (Montembault et al., 2017). While the chromatin-associated signal is not known, a likely candidate is Ran GTPase.

Active Ran forms an inverse gradient with importins to control ring positioning (Figure 2C; Kiyomitsu and Cheeseman, 2013; Beaudet et al., 2017; Beaudet et al., 2020). Importin-α and -β bind to nuclear localization signals (NLSs) in proteins and Ran-GTP dissociates this complex (Xu and Massague, 2004; Lange et al., 2007; Clarke and Zhang, 2008; Ozugergin and Piekny, 2021). Ran-GTP is generated by histone-tethered RCC1 (RanGEF), while cytosolic RanGAP negatively regulates Ran, causing active Ran to be highest around chromatin and lowest near the cortex (Figure 1B; Kalab et al., 2002; Kalab et al., 2006). In anaphase, the segregating chromosomes could lead to the equatorial enrichment of importins where they control the localization and function of anillin (Hinkle et al., 2002; Kiyomitsu and Cheeseman, 2013; Beaudet et al., 2017). In meiosis, active Ran functions as a ruler to control formation of an F-actin cap for polar body extrusion in mouse oocytes (Deng et al., 2007). Although the cortical targets of Ran signaling in meiosis are not known, they regulate branched F-actin (Yi et al., 2011; Dehapiot et al., 2013; Burdyniuk et al., 2018). Importins also regulate cellularization of the syncytial *Drosophila* embryo, where ingressing membranes partition nuclei into individual cells (Lecuit, 2004). Silverman-Gavrila et al. (2008) showed that importin-α overexpression causes a decrease in anillin and Peanut (*Dm*Septin) localization and prevents cellularization, because importins compete with Peanut for anillin-binding. Importin-β overexpression also decreases anillin’s cortical localization in HeLa cells, supporting the ruler model where different levels of importins promote or inhibit function. This model is supported by the molecular regulation of anillin; the RhoA-GTP binding domain autoinhibits a neighbouring domain with overlapping NLS and phospholipid-binding sites, and RhoA-GTP relieves this autoinhibition, permitting importin-binding to stabilize anillin for recruitment to the overlying phospholipids (Beaudet et al., 2017; Beaudet et al., 2020). We propose that importins are sufficiently enriched only between the segregating chromosomes in cells where Ran-GTP reaches the cortex (e.g., higher ploidy; Figure 2D), while in cells where cortical importins are uniform, other mechanisms would control ring positioning (e.g., lower ploidy; Figure 2E).

The Ran pathway could control cortical targets other than anillin (Ozugergin et al., 2022). In *C. elegans* embryos, importin-β (IMB-1) facilitates the equatorial enrichment of ANI-1 in a somatic cell, while importin-α (IMA-3) and/or -β control ring assembly in a germline-fated cell through unknown targets. Also, importins could bind as homo- or heterodimers which could differently impact protein function (Ozugergin and Piekny, 2021). An exciting hypothesis is that the Ran pathway has multiple targets that respond to different importin levels to confer the cortical properties controlling cytokinesis in diverse cell types.

## Discussion

After a century of research, our understanding of cytokinesis is extensive. However, there is considerable diversity in how the core machinery is expressed and regulated, and in the number of mechanisms that control cytokinesis. The differences we reviewed here are just the tip of the iceberg, reflecting the need to break away from the ‘one-size-fits-all’ approach. Novel research exploring differences among diverse cell types is crucial to reveal how cytokinesis can be ‘personalized’, and to gain an appreciation of its diversity.
